# Determination of Photosensitizing Potential of Lapachol for Photodynamic Inactivation of Bacteria

**DOI:** 10.3390/molecules29215184

**Published:** 2024-11-02

**Authors:** Regiane G. Lima, Raphael S. Flores, Gabriella Miessi, Jhoenne H. V. Pulcherio, Laís F. Aguilera, Leandro O. Araujo, Samuel L. Oliveira, Anderson R. L. Caires

**Affiliations:** Optics and Photonics Group, Institute of Physics, Federal University of Mato Grosso do Sul, P.O. Box 549, 79070-900 Campo Grande, MS, Brazil; regigodoy@gmail.com (R.G.L.); raphael.rflores@gmail.com (R.S.F.); gabriella.miessi@ufms.br (G.M.); jhoenne_vasconcelos@ufms.br (J.H.V.P.); lais_fernandes13@hotmail.com (L.F.A.); leandro.oliveira22@outlook.com (L.O.A.); samuel.oliveira@ufms.br (S.L.O.)

**Keywords:** lapachol, blue light, antimicrobial photodynamic inactivation, bacteria

## Abstract

Antimicrobial photodynamic inactivation (aPDI) offers a promising alternative to combat drug-resistant bacteria. This study explores the potential of lapachol, a natural naphthoquinone derived from *Tabebuia avellanedae*, as a photosensitizer (PS) for aPDI. Lapachol’s photosensitizing properties were evaluated using *Staphylococcus aureus* and *Escherichia coli* strains under blue LED light (450 nm). UV-vis spectroscopy confirmed lapachol’s absorption peak at 482 nm, aligning with effective excitation wavelengths for phototherapy. Photoinactivation assays demonstrated significant bacterial growth inhibition, achieving complete eradication of *S. aureus* at 25 µg·mL^−1^ under light exposure. Scanning electron microscopy (SEM) revealed morphological damage in irradiated bacterial cells, confirming lapachol’s bactericidal effect. This research underscores lapachol’s potential as a novel photosensitizer in antimicrobial photodynamic therapy, addressing a critical need in combating antibiotic resistance.

## 1. Introduction

Since the late 1920s, following the discovery of penicillin, antibiotics have been integral to surgery, transplants, and critical care [1]. However, their widespread use has led to the rise in drug-resistant strains, marking a return to the pre-antibiotic era [2]. The options for treating drug-resistant infections are extremely limited, often forcing the use of toxic drugs previously abandoned [3]. By 2050, it is estimated that antibiotic-resistant microorganisms could cause over 10 million deaths annually if no action is taken [4]. This scenario suggests that resistant bacteria may surpass cancer in causing deaths, with an estimated economic cost of USD 100 trillion [5].

Bacteria are usually classified by their shape (bacilli, cocci, or spiral) or their ability to retain Gram stains (Gram-positive or Gram-negative). This latter classification highlights differences in cellular structure. Gram-positive bacteria have a simpler structure, consisting only of a cell wall, whereas Gram-negative bacteria possess an additional outer membrane, providing extra protection and making them more challenging to treat [6].

*Escherichia coli* (*E. coli*), a Gram-negative bacterium, is a common cause of sepsis and hospital-acquired infections in humans and animals [7]. *Staphylococcus aureus* (*S. aureus*), a Gram-positive bacterium, is responsible for food poisoning, skin infections, and pneumonia [8]. Both bacteria have developed resistance to multiple antibiotics and are spreading rapidly due to poor sanitation and hygiene in communities and hospitals [7,8].

In response to this challenge, antimicrobial photodynamic inactivation (aPDI) has emerged as a promising alternative to combat bacterial infections. This technique combines a photosensitizer (PS), light, and molecular oxygen (O_2_). The PS absorbs light and transfers energy or charge to oxygen, transforming its stable triplet state (^3^O_2_) into reactive oxygen species (ROS) or singlet oxygen (^1^O_2_) through type I and type II mechanisms, respectively [9]. In type I, the PS transfers an electron to oxygen, producing free radicals and peroxides, while in type II, energy is transferred, transforming ^3^O_2_ into ^1^O_2_ [10].

The interest in aPDI increased when it was established that aPDI can be effective regardless of bacterial resistance [11]. Additionally, it is not expected that aPDI triggers bacterial resistance because of its multi-site mode of action [11]. Consequently, there is a growing search for new molecules and materials to act as antimicrobial photosensitizers (aPSs) [10]. Among them, natural-based aPSs have attracted interest. Derived from natural sources like plants, algae, or microorganisms, they are usually more compatible with the biological environment [12], presenting reduced toxicity and being well tolerated by the human body [12].

Chlorophyll is a natural photosensitizer (PS) found in green plants [13], while curcumin and natural quinones are also promising due to their ability to absorb light and generate ROS [14,15]. Quinones, with their redox properties, play key roles in plants, including defense, oxidative phosphorylation, and redox signaling [15,16,17,18].

Lapachol, a naphthoquinone, is commonly extracted from the pink Ipe tree (*Tabebuia avellanedae*), a species in the Bignoniaceae family and one of the most beautiful trees in the Brazilian flora. This molecule has various pharmacological activities, including antimicrobial, antifungal, molluscicidal, leishmanicidal, antibacterial, trypanocidal, antimalarial, anti-inflammatory, anticancer, antiulcer, contraceptive, and immunosuppressive effects [16,19,20,21,22].

Lapachol was first described by Paternò in 1882 with a chemical structure elucidated in 1896 by Hooker, who identified it as a naphthoquinone, specifically 2-hydroxy-3-(e-methyl-2-butenyl)-1,4-naphthoquinone [19]. The biological activities attributed to lapachol, along with some of its natural and synthetic derivatives, have increased the importance of this class of substances as promising candidates in the search for new drug development [23,24].

Studies have explored the antibacterial properties of lapachol, its derivatives, and other naphthoquinones, suggesting potential antimicrobial activity against bacteria strains [25,26,27]. Investigations on the minimal inhibitory concentration (MIC) for *S. aureus* reported high values, ranging from 128 to 256 µg.mL^−1^ (1–4). In contrast, the MIC for *Escherichia coli* demonstrated no toxicity, even at elevated concentrations and higher doses [22,25]. Despite its numerous bioactivities, there is a lack of studies exploring the use of lapachol in antimicrobial photodynamic inactivation. However, it is important to point out that that some studies have already explored the use of quinones in photodynamic therapy (PDT), primarily in the context of cancer treatment [27,28,29,30,31,32]. Naphthoquinones, such as lapachol and its derivatives, have been investigated in PDT, particularly in the 700 nm irradiation region [27]. However, there is a lack of research on the application of quinones and naphthoquinones in antimicrobial photodynamic inactivation (aPDI), a gap that will be addressed in the present work.

## 2. Results

### 2.1. Optical Characterization of Lapachol

**Figure 1:**
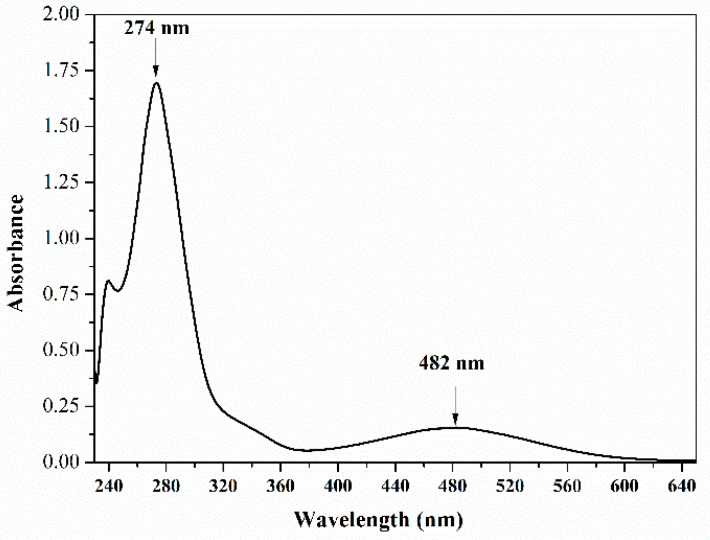
UV-vis absorption spectrum of lapachol at 25 µg·mL^−1^ in PBS solution at pH 7.

Figure 1 presents a typical UV-vis absorption spectrum of lapachol in PBS at pH 7.0. Two prominent absorption bands exist, centered at around 280 and 480 nm, in the UV and visible range, respectively. This spectrum profile agrees with the observed electronic spectra of substituted naphthoquinones, in which a strong π → π* transition of the quinone ring is expected at around 277 nm and a low-intensity band, in the 400 to 500 nm, can be assigned to the n → π* transition of the quinone carbonyls [33,34].

**Figure 2:**
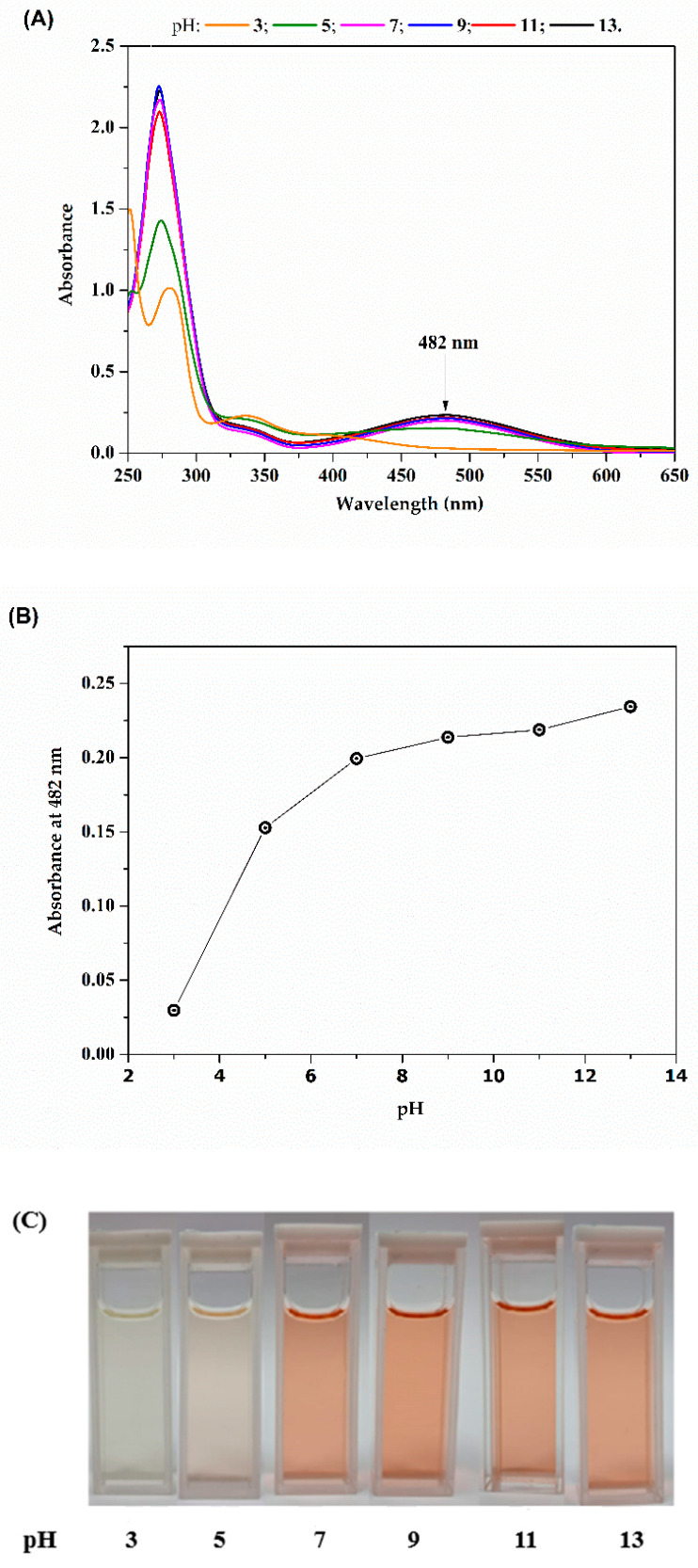
(**A**) UV-vis absorption spectra of lapachol at 20 µg·mL^−1^ for different pHs, (**B**) lapachol absorbance at 482 nm and (**C**) images of lapachol with different pHs.

Figure 2A presents the absorption spectra of lapachol when subjected to different pHs. Our results are in accordance with previous observations, in which a distinct color change occurs in an aqueous solution, transitioning from intense red under alkaline conditions to yellow when the pH drops below 5–6 [35]. Under acid conditions, lapachol precipitates as a yellow powder [35]. This is a consequence of the weak acid behavior of lapachol due to the presence of a hydroxyl group in its molecular structure. Our observation confirms not only the relation between the pH and different absorption bands but also indicates that, for more alkaline solutions, the band at 482 nm is far more expressive compared to the more acid ones. In fact, the peak centered at 482 nm disappears around pH 5, coinciding with the initiation of precipitation.

As previously discussed by Segoloni and Di Maria [35], the electronic pair, previously engaged in the hydrogen bond within the associated form, contributes to enhancing the –O− auxochrome action in relation to the adjacent chromophore group C=O in alkaline conditions. This interaction leads to an increase in absorbance at higher wavelengths, particularly around 482 nm, as the lone pair engages with the quinoid ring. The likelihood is that intermolecular interactions between the lone pairs of hydroxyl anions in the solution and chromophore groups C=O also play a significant role. This is supported by the observation that compounds like lapachol, such as naphthoquinones lacking hydroxyl groups, yield reddish alkaline solutions, suggesting a distinct influence of intermolecular interactions in such systems [35].

The outcomes demonstrate that lapachol, when in an alkaline solution, presents a wide absorption band in the visible region (from 400 to 600 nm), one of the desirable characteristics for a potential photosensitizer in photodynamic therapy. Additionally, its solubility in water is notably influenced by pH, ranging from 2.5 µg·mL^−1^ at pH 4 to 5 mg·mL^−1^ at pH 10.0 [35]. It readily dissolves in alkaline aqueous solutions, diethyl ether, acetone, dichloromethane, and petroleum ether; it is also highly soluble in methanol, ethanol, chloroform, and benzene [35].

### 2.2. Production of Reactive Oxygen Species (ROS)

**Figure 3:**
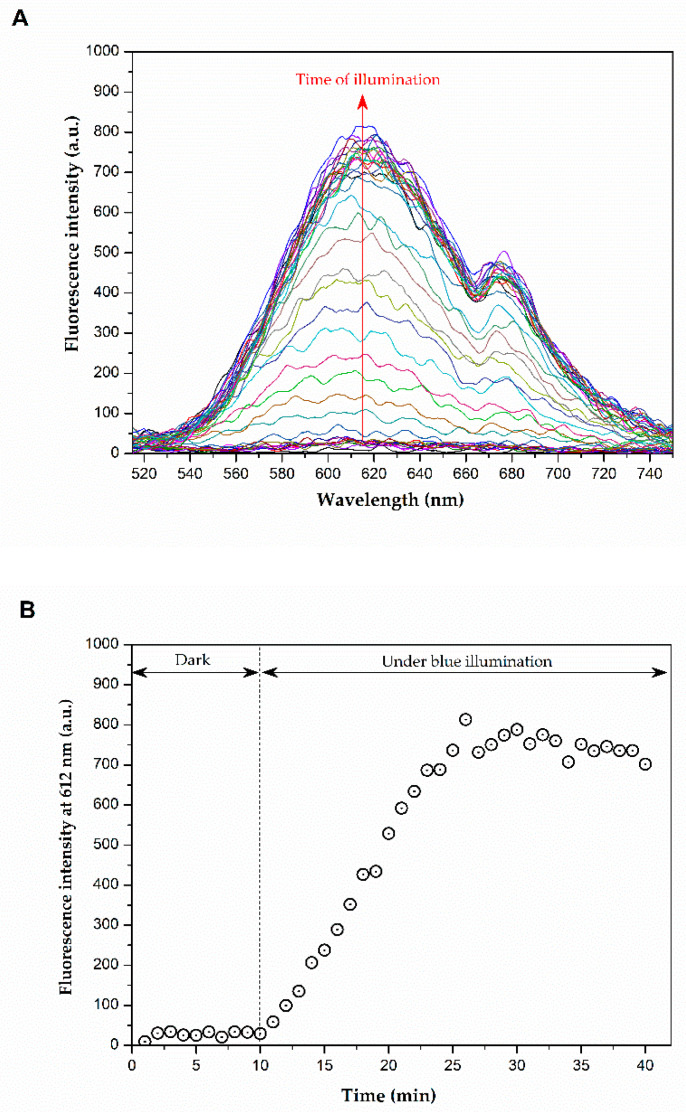
(**A**) Fluorescence spectra of DHE in the presence of lapachol and (**B**) fluorescence intensity at 612 nm as function of illumination time.

Figure 3A displays the fluorescence spectra of a mixed solution of lapachol and DHE when kept in the dark and when exposed to blue light irradiation. The results indicate that no fluorescence is detected when the sample is kept in the dark, as shown in Figure 3B. In contrast, during illumination, an increase in fluorescence signal over time is observed, which is attributed to the generation of reactive oxygen species (ROS). This occurs because dihydroethidium (DHE), a non-fluorescent molecule, produces a fluorescent product (ethidium) upon interacting with various ROS species, such as hydrogen peroxide (H_2_O_2_), superoxide (O^2−^), hypochlorous acid (HOCl), and peroxynitrite (ONOO^−^) [36]. These data confirm that lapachol are capable of generating ROS under blue light illumination.

### 2.3. Photoinactivation Assay

**Figure 4:**
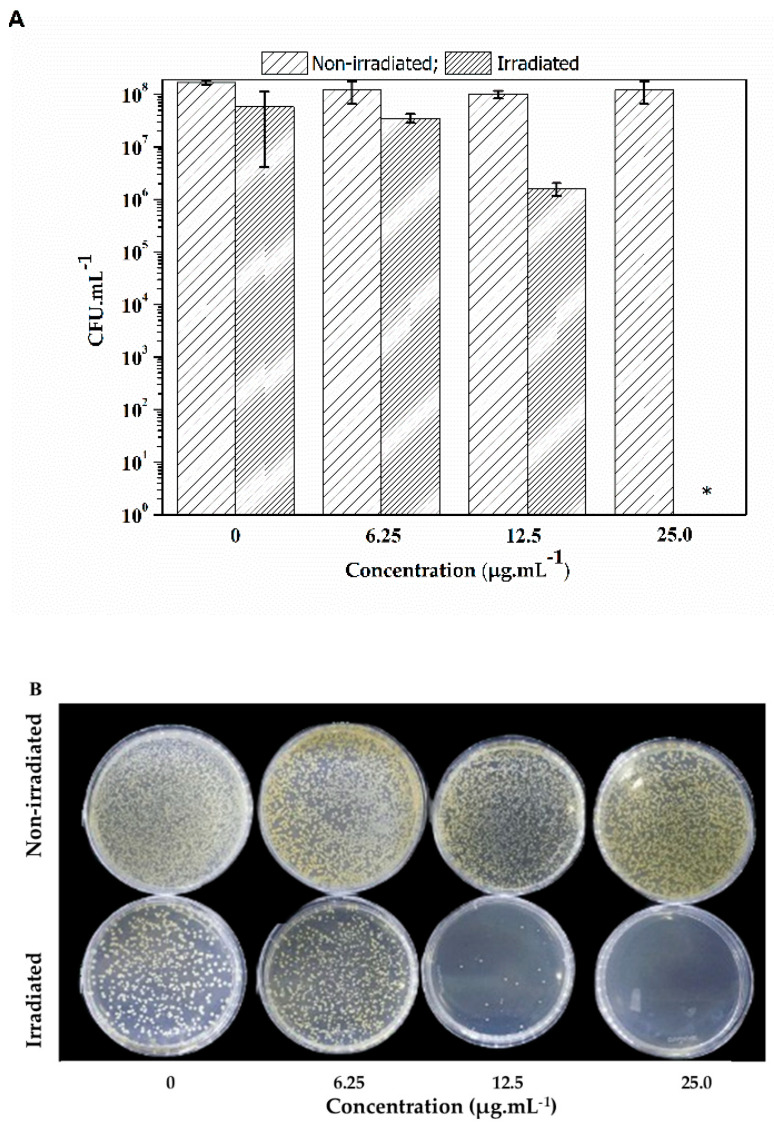
(**A**) CFU mean values (±SD) of *S. aureus* with lapachol for non-irradiated and irradiated under 450 nm and 28 mW cm^−2^ for 60 min. (**B**) Growth of *S. aureus* colonies in Petri dishes containing negative control 6.25, 12.5, and 25.0 µg/mL of lapachol. The bacterial suspension was illuminated at 450 nm with an energy dose of 0 (non-irradiated) and 100 J cm^−2^ (irradiated). * Indicates no colony growth under irradiation in this concentration.

Figure 4 shows representative images of *S. aureus* colonies (strains ATCC and mcr-1) subjected to lapachol at 0.0, 6.5, 12.5, and 25.0 µg·mL^−1^ when kept in the dark (non-irradiated) and illuminated by blue light (450 nm) at an energy dose of 100 J·cm^−2^ for 60 min. Lapachol can effectively inactivate *S. aureus* using concentrations over 12.5 µg·mL^−1^. The data also revealed that lapachol alone could not inhibit the development of the non-irradiated colonies.

**Figure 5:**
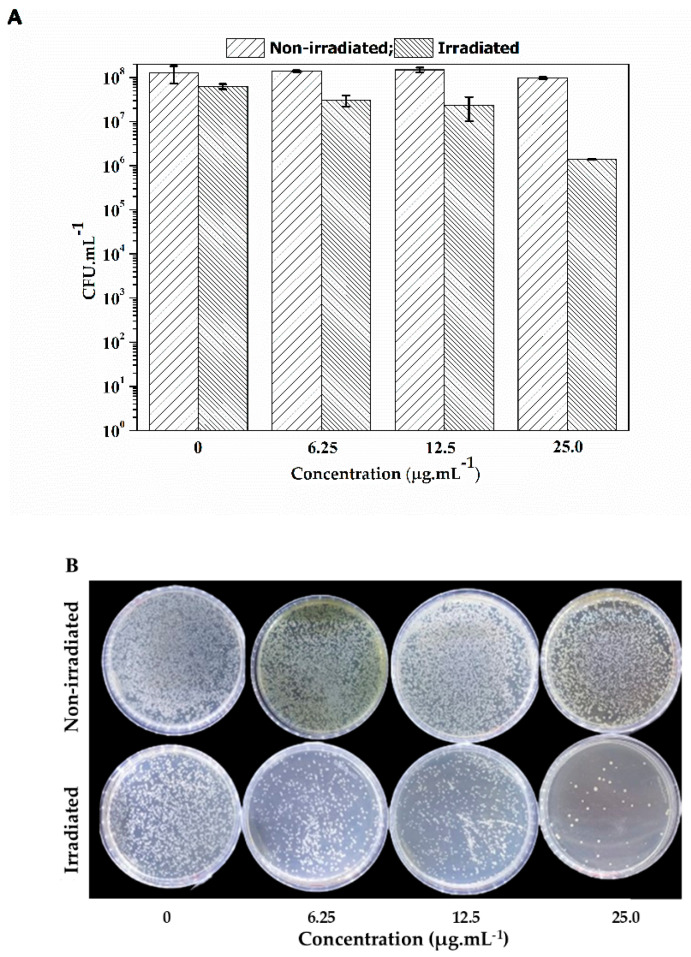
(**A**) CFU mean values (±SD) of *E. coli* with lapachol for non-irradiated and irradiated under 450 nm and 28 mW·cm^−2^ for 90 min. (**B**) Growth of *E. coli* colonies *in Petri* dishes containing negative control 6.25, 12.5, and 25.0 µg·mL^−1^ of lapachol. The bacterial suspension was non-irradiated and irradiated at 450 nm with an energy dose of 150 J·cm^−2^.

Initially, the *E. coli* strain was also subjected to the same energy dose (100 J cm^−2^). However, no inhibition of bacterial growth was observed. Consequently, lapachol is less efficient as a PS against *E. coli* when compared with *S. aureus*. Figure 5 reveals that even when using a higher light dose than that applied against *S. aureus*, a maximum of two-log reduction was observed for a combination of lapachol at 25.0 µg·mL^−1^ under a blue light illumination at 28 mW·cm^−2^ for 90 min, totaling 150 J·cm^−2^.

It is known that, in general, Gram-negative bacteria have more protection structures against PS internalization and the ROS generated in photodynamic inactivation when contrasted with Gram-positive bacteria. It is usually caused by the presence of a highly organized outer membrane in the cell wall of Gram-negative bacteria [37,38].

Some studies have overcome this problem by choosing a longer incubation period and adding a permeabilization agent to provide a more efficient photoinactivation [39]. In the present study, DMSO at a 0.1% (v.v^−1^) concentration was present in both *E. coli* and *S. aureus* assay and acted as a permeabilization agent. However, the initial goal was to use DMSO as a solvent for lapachol, since it does not naturally solubilize in an aqueous medium.

It is important to highlight that lapachol proved to be non-toxic in the non-irradiated group for both *E. coli* and *S. aureus* assays in the tested concentrations, emphasizing that lapachol is a photo-activated agent for the photodynamic inactivation process.

SEM was used to analyze morphological changes in bacteria after being subjected to aPDI. The concentration of lapachol used was 25 µg·mL^−1^, for both groups.

**Figure 6:**
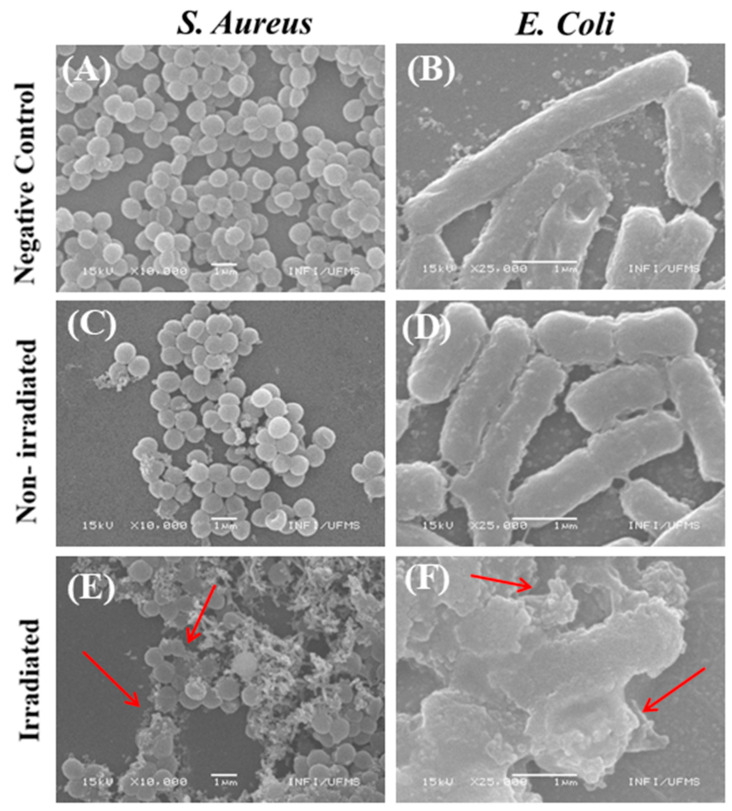
Representative SEM images of *S. aureus* and *E. coli*, respectively, subjected to the (**A**,**B**) negative control, lapachol at 25 µg·mL^−1^ kept in the dark; (**C**,**D**) non-irradiated group; and irradiated (**E**,**F**) with a blue light (450 nm) at 28 mW cm^−2^ during 60 min for *S. aureus* and 90 min for *E. coli*. Red arrows indicate partial or complete damage to the cell wall. All scale bars represent a length of 1 µm.

The bacterial cells presented a smooth and intact surface, without morphological changes or apparent cell lysis when subjected to the negative control groups, as shown in Figure 6A,B. The data showed the *S. aureus* in the shape of cocci gathered in clusters, with a diameter of approximately 1 μm, and *E. coli* in the form of rods, with a diameter close to 0.5 and 3 μm in length, both presenting normal morphology. For the bacteria subjected to the lapachol without irradiation, the findings revealed no membrane damage, as presented in Figure 6C,D.

In contrast, the photodynamic inactivation process led to significant changes, resulting in bacterial lysis due to partial or complete damage to the bacterial cell wall of *S. aureus* and *E. coli* when exposed to a light dose of 100 and 150 J·cm^−2^, respectively (Figure 6E,F).

## 3. Discussion

Lapachol, a naphthoquinone extracted from the pink Ipe tree, has demonstrated a range of bioactivities, including antimicrobial and anticancer effects. In general, quinone metabolites perform various essential functions in plants, such as defense pathogen, involvement in oxidative phosphorylation, and contributions to redox signaling [15,16]. Quinones found in plants can be classified into benzoquinones, naphthoquinones, anthraquinones, and phananthraquinones, depending on the type of aromatic system [15,16,17]. The defining characteristic of quinones is their redox property, driven by the forming of aromatic systems. Thus, these compounds can participate in multiple biological oxidative processes due to their structural properties [18]. The antitumor mechanism of these compounds, for example, is based on a redox cycle that generates ROS [17].

Natural-based quinones have been pointed out as promising aPSs due to their capacity to generate ROS [15]. Quinone-based PSs have proven helpful in photodynamic therapy (PDT) for treating cancer [22]. These compounds can generate ROS and efficiently produce ^1^O_2_, making them effective agents for targeting and destroying cancer cells. To illustrate, Guo et al. developed customized photosensitizers by combining 9,10-phenanthrenequinone (PQ) with electron-donating triphenylamine derivatives. This unique combination showed exceptional type I ROS generation and efficient photothermal conversion capabilities [28]. Quinoxalinone CN (QCN), another quinoxalinone-based photosensitizer, produces ^1^O_2_ under 530 nm irradiation. Additionally, QCN exhibited aggregation-induced near-infrared (NIR), making it a promising candidate for image-guided PDT [33].

Jadhao et al. investigated the interaction of aluminum phthalocyanine tetrasulphonate (AlPcS4), a quinone-based photosensitizer, with DNA alkylating quinone in biomimicking micellar microenvironments for PDT applications [30].

Lapachol can be strategically modified to create fluorescent compounds with unique properties. Rodrigues et al. synthesized a novel trans-A2B-corrole derivative from lapachol [31]. Its characterization revealed remarkable fluorescence and optical absorption characteristics. Photobiological assays confirmed the compound’s photostability, singlet oxygen production, and effective ROS generation, suggesting suitability for PDT [31].

Campanholi et al. evaluated the efficacy of pheophorbide and zinc-pheophorbide photosensitizers combined with lapachol and β-lapachone drugs in biocompatible nanocarriers Pluronic P123 and F127. Spectrophotometric analysis revealed monomerization of the photosensitizer within the formulation. Lapachol encapsulated in copolymeric micelles showed slight pKa variations. β-lapachone systems exhibited reduced fluorescence and singlet oxygen quantum yields. A five-fold increase in singlet oxygen lifetime indicated enhanced stability in the nanostructured microenvironment. Combined systems demonstrated thermal stability, even in a lyophilized state, maintaining a monomeric photosensitizer state, with most showing temporal stability beyond 96 h. However, limitations included changes in carrier system properties during freezing, drugs unrecoverable after lyophilization, and potential degradation processes of photosensitizers. The lapachol and β-lapachone formulation stabilized photosensitizers, inducing increased micellar segment hydration, reducing quantum yields, and enhancing temporal stability. Marked thermal reversibility and a high singlet oxygen lifetime in the micellar microenvironment suggested suitability for practical photodynamic therapy (PDT) applications, potentially complementing clinical treatments and expanding application possibilities [32].

However, some studies have already explored the use of quinones in PDT, primarily in the context of cancer treatment. The present study demonstrates the promising potential of lapachol to be used in aPDI. The optical characterization of lapachol reveals its pH-dependent behavior, with UV-vis absorption spectra showing notable absorption bands at 280 nm and 480 nm. These bands correspond to the π → π* transition and n → π* transition of the quinone ring, respectively. The absorption spectrum changes with pH, with a red color observed in alkaline conditions and a yellow precipitate forming in acidic conditions. This indicates that the peak at 482 nm intensifies in alkaline solutions but disappears as the pH decreases below 5. The ability of lapachol to absorb in the visible region (400–600 nm) and its solubility at different pH levels make it a promising candidate for use as a photosensitizer in photodynamic therapy. In fact, the efficacy of lapachol as a PS was demonstrated through its ability to generate reactive oxygen species (ROS) under blue light illumination, which was confirmed by the photoinactivation assays. Lapachol effectively inactivates *S. aureus* at concentrations over 12.5 µg/mL when exposed to light, while it had no significant effect on bacterial growth in non-irradiated conditions. However, lapachol was less effective against *E. coli*, even under higher energy doses, which may be due to the structural differences in the cell walls of Gram-negative bacteria, providing more protection.

## 4. Materials and Methods

### 4.1. Photosensitizer

The lapachol standards purity grade was ≥98%, 2-Hydroxy-3-(3-methyl-2-butenyl)-1,4-naphthoquinone (Sigma-Aldrich, St. Louis, MO, USA) CAS: 84-79-7.

Lapachol was diluted in dimethyl sulfoxide (DMSO, Sigma-Aldrich, St. Louis, MO, USA) at 2.5 mg.mL^−1^ to obtain a stock solution. Then, the tested solutions of lapachol were prepared in phosphate-buffered saline (PBS, Sigma-Aldrich, St. Louis, MO, USA) at different concentrations.

### 4.2. UV-Vis Absorption

UV-vis spectrophotometry was performed in the 200–700 nm range in a PerkinElmer (Waltham, MA, USA), model Lambda 265, device. UV-vis absorption spectra of lapachol in PBS solution were collected at room temperature.

Buffer solutions were prepared in pH levels of 3, 5, 7, 11, and 13 to perform UV-vis spectrophotometry of lapachol (originally in an alkaline mixture) dissolutions in different pH levels. The base buffer mixture was obtained by solubilizing 1.2 g of sodium citrate dihydrate (Sigma-Aldrich, St. Louis, MO, USA) and 1.1 g of citric acid (Sigma-Aldrich, St. Louis, MO, USA) in 1 L of distilled water. After the solubilization, sodium hydroxide was utilized to increase the pH to the desired values.

### 4.3. Determination of Reactive Oxygen Species Production

The determination of reactive oxygen species (ROS) was carried out as described by Caires et al. with adaptations [36]. A total of 140 µL of DHE at 5 mM was added into 2.0 mL of an aqueous solution of lapachol at 25 µg·mL^−1^. Initially, the sample was kept in the dark during 10 min and then exposed to blue light (450 nm) at 28.82 mW·cm^−2^ for 30 min. The fluorescence spectra were collected in the 520–750 nm range, every 1 min, by exciting at 500 nm with the aid of a spectrofluorometer (FluoroMate FS-2, Sinco, Seoul, Republic of Korea).

### 4.4. Photoinactivation Assay

The aPDI assays were tested against *Staphylococcus aureus* strain ATCC 25923 and *Escherichia coli* strain ATCC 25922. The strains were stored at 2 °C in Müller Hinton Broth (Sigma-Aldrich, St. Louis, MO, USA) enriched with glycerol in 20%. To prepare the bacterial suspensions, 40 µL of each bacterial strain was inoculated into 2 mL of BHI medium (brain heart infusion) and incubated at 37 °C for 24 h. Subsequently, the bacterial concentration was adjusted to reach 0.5 on the McFarland scale by adding phosphate-buffered saline (PBS) until it reached an absorbance of around 0.14 at 625 nm with the aid of an Agilent BioTek Synergy H1 Multimode Reader (Santa Clara, CA, USA).

The lapachol concentrations were obtained by diluting the stock solution in 2 mL of saline solution containing the bacterial inoculum and a permeabilization agent, dimethyl sulfoxide (DMSO) at a concentration of 0.1% (v·v^−1^). The tested concentrations of lapachol ranged from 0.0 (negative control) to 25 µg·mL^−1^. Following the addition of the lapachol, the samples underwent agitation at 120 rpm for 60 (or 90) min in a shaker (Marconi, Piracicaba, Brazil) before irradiation. Then, the samples were divided into two distinct groups; one was subjected to light (called the irradiated group), while the other group was kept in darkness (the non-irradiated group). The irradiated group was exposed to blue light irradiation (450 nm), using light-emitting diodes (LEDs), at 28 mW·cm^−2^ for 60 or 90 min, depending on the bacterial strain.

Finally, both groups (irradiated and non-irradiated) had 200 µL of each sample concentration dispensed into a 96-well microplate. Subsequently, a serial dilution was executed until reaching a 1:32 ratio. The total bacterial count was determined using the spread plate method in a plate count agar (PCA) medium. Colony-forming units (CFU) were counted after 18 h of incubation at 37 °C. All measurements were performed in duplicate and repeated three times to determine the CFU mean values (±SD). Quantitative and statistical analyses were performed using Origin 8.5, considering the repetitions and treatments. This analysis encompassed CFU values obtained for the 4 concentrations of photosensitizer, the irradiated and dark control groups, and the two strains of bacteria (*E. coli* and *S. aureus*).

### 4.5. Scanning Electron Microscopy (SEM)

Before sample preparation, an aPDI bioassay was conducted, following the procedure outlined in Section 2.3, to photoinactivation *E. coli* and *S. aureus* strains. The experiment was divided into two groups: one subjected to irradiation and another kept in the dark. Two samples were prepared for each group—one as a negative control (without lapachol) and another containing lapachol at 25 µg·mL^−1^.

The process began with the distribution of 200 µL of irradiated and dark-kept samples into Eppendorf tubes, followed by adding of 1 mL of 2.5% glutaraldehyde PBS solution. After the 3 h period, the samples were centrifuged at 1000 rpm for 5 min. Subsequently, 1 mL was removed, and 1 mL of PBS was added; this procedure was repeated thrice.

For dehydration, ethanol solutions at 25%, 50%, 70%, 80%, 90%, and 100% were used. The process involved removing 1 mL of PBS and adding 1 mL of ethanol concentration, starting with 25%. After each step, the samples were centrifuged for 5 min at 1000 rpm. This procedure was repeated for all ethanol concentrations until it reached 100%.

Upon completion of the process, 10 µL of the samples was added to silicon surfaces and left to dry and fixed at room temperature for 24 h. Finally, scanning electron microscopy (SEM) images were collected using an SEM microscope (JEOL, model JSM-6380LV, Akishima, Japan) with an accelerating voltage of 15 kV.

## 5. Conclusions

In conclusion, this study highlights the significant potential of lapachol as a potential photosensitizer for aPDI against Gram-positive and Gram-negative bacteria. Nevertheless, although lapachol reduced bacterial growth for both bacteria, it demonstrated lower efficacy in the *E. coli* strain (Gram-negative strain) compared to *S. aureus* (Gram-positive strain). While no bacterial growth was observed when *S. aureus* was exposed to lapachol at 25.0 µg·mL^−1^ under blue light irradiation of 100 J·cm^−2^, only a two-log reduction was achieved for *E. coli* even when applying a higher blue light dose (150 J·cm^2^) with lapachol at the same concentration (25.0 µg·mL^−1^). Additionally, our SEM data confirmed that aPDI action promoted by lapachol significantly damaged the bacterial cell wall, lysing the bacterial cells. Overall, this study provides fundamental insight into the potential use of lapachol as a natural-based photosensitizer in antimicrobial photodynamic therapy, addressing an urgent need for natural-based therapeutic materials in modern medicine.

## Data Availability

Data available on request.

## References

[1] Runcie H. (2015). Infection in a Pre-Antibiotic Era. J. Anc. Dis. Prev. Remedies.

[2] Arias C.A., Murray B.E. (2009). Antibiotic-Resistant Bugs in the 21st Century—A Clinical Super-Challenge. New Engl. J. Med..

[3] Boucher H.W., Talbot G.H., Bradley J.S., Edwards J.E., Gilbert D., Rice L.B., Scheld M., Spellberg B., Bartlett J. (2009). Bad Bugs, No Drugs: No ESKAPE! An Update from the Infectious Diseases Society of America. Clin. Infect. Dis..

[4] Tang K.W.K., Millar B.C., Moore J.E. (2023). Antimicrobial Resistance (AMR). Br. J. Biomed. Sci..

[5] O’Neill J. (2016). Review on Antimicrobial Resistance: Tackling Drug-Resistant Infections Globally: Final Report and Recommendations.

[6] Baron S. (1996). Medical Microbiology.

[7] Laxminarayan R., Duse A., Wattal C., Zaidi A.K.M., Wertheim H.F.L., Sumpradit N., Vlieghe E., Hara G.L., Gould I.M., Goossens H. (2013). Antibiotic Resistance—The Need for Global Solutions. Lancet Infect. Dis..

[8] Todd E.C.D. (2014). Bacteria: Staphylococcus Aureus. Encyclopedia of Food Safety.

[9] Sperandio F., Huang Y.-Y., Hamblin M. (2013). Antimicrobial Photodynamic Therapy to Kill Gram-Negative Bacteria. Recent. Pat. Antiinfect. Drug Discov..

[10] Hamblin M.R., Hasan T. (2004). Photodynamic Therapy: A New Antimicrobial Approach to Infectious Disease?. Photochem. Photobiol. Sci..

[11] Liu Y., Qin R., Zaat S.A., Breukink E., Heger M. (2015). Antibacterial Photodynamic Therapy: Overview of a Promising Approach to Fight Antibiotic-Resistant Bacterial Infections. J. Clin. Transl. Res..

[12] Vilaça R.M.A., Sodré G.P., Ferreira I.S., Da Costa G.D., Dias L.D. (2023). Fotossensibilizadores de Origem Natural: Extração, Caracterização e Recentes Avanços Na Fotoinativação Bacteriana. Braz. J. Health Rev..

[13] Ludačka P., Kubát P., Bosáková Z., Mosinger J. (2022). Antibacterial Nanoparticles with Natural Photosensitizers Extracted from Spinach Leaves. ACS Omega.

[14] Dias L.D., Blanco K.C., Mfouo-Tynga I.S., Inada N.M., Bagnato V.S. (2020). Curcumin as a Photosensitizer: From Molecular Structure to Recent Advances in Antimicrobial Photodynamic Therapy. J. Photochem. Photobiol. C Photochem. Rev..

[15] Rajendran M. (2016). Quinones as Photosensitizer for Photodynamic Therapy: ROS Generation, Mechanism and Detection Methods. Photodiagnosis Photodyn. Ther..

[16] Hussain H., Krohn K., Ahmad V.U., Miana G.A., Green I.R. (2007). Lapachol: An Overview. Arkivoc.

[17] Silva C.C., Oliveira Paiva R., Costa G.L., Echevarria A. (2021). Anti-Bacterial Activity of New 2-Amino-1, 4-Naphthoquinones. Braz. J. Dev..

[18] Januário S.R., Silvério-lopes S. (2014). O Poder Terapêutico Do Ipê Roxo e Seu Uso Na Terapia Complementar Ao Tratamento de Neoplasias. Rev. Bras. Saúde.

[19] Araújo E.L., Alencar J.R.B., Neto P.J.R. (2002). Lapachol: Segurança e Eficácia Na Terapêutica. Rev. Bras. Farmacogn..

[20] Lira A.A.M., Sester E.A., Carvalho A.L.M., Strattmann R.R., Albuquerque M.M., Wanderley A.G., Santana D.P. (2008). Development of Lapachol Topical Formulation: Anti-Inflammatory Study of a Selected Formulation. Am. Assoc. Pharm. Sci..

[21] Chen Q., Bai L., Zhou X., Xu P., Li X., Xu H., Zheng Y., Zhao Y., Lu S., Xue M. (2020). Development of Long-Circulating Lapachol Nanoparticles: Formation, Characterization, Pharmacokinetics, Distribution and Cytotoxicity. RSC Adv..

[22] Linzner N., Fritsch V.N., Busche T., Tung Q.N., Loi V.V., Bernhardt J., Kalinowski J., Antelmann H. (2020). The Plant-Derived Naphthoquinone Lapachol Causes an Oxidative Stress Response in Staphylococcus Aureus. Free Radic. Biol. Med..

[23] Nepomuceno J.C. (2014). Lapachol and Its Derivatives as Potential Drugs for Cancer Treatment. Plants and Crop the Biology and Biotechnology Research.

[24] Salustiano E.J.S., Netto C.D., Fernandes R.F., Silva A.J.M.d., Bacelar T.S., Castro C.P., Buarque C.D., Maia R.C., Rumjanek V.M., Costa P.R.R. (2010). Comparison of the Cytotoxic Effect of Lapachol, α-Lapachone and Pentacyclic 1, 4-Naphthoquinones. Investig. New Drugs.

[25] Souza M.A., Johann S., Alves L., Lima S., Campos F.F., Mendes I.C., Beraldo H., Souza-fagundes E.M.D., Cisalpino P.S., Rosa C.A. (2013). The Antimicrobial Activity of Lapachol and Its Thiosemicarbazone and Semicarbazone Derivatives. Mem. Inst. Oswaldo Cruz.

[26] Oliveira C.G.T., Miranda F.F., Ferreira V.F., Freitas C.C., Rabello R.F., Carballido J.M., Corrêa L.C.D. (2001). Synthesis and Antimicrobial Evaluation of 3-Hydrazino-Naphthoquinones as Analogs of Lapachol. J. Braz. Chem. Soc..

[27] Eyong K.O., Kumar S.P., Kuete V., Folefoc G.N., Langmi H., Meyer M.J.J., Lall N., Baskaran S. (2012). Cobalt Mediated Ring Contraction Reaction of Lapachol and Initial Antibacterial Evaluation of Naphthoquinones Derived from Lapachol. Med. Chem. Res..

[28] Guo J., Dai J., Peng X., Wang Q., Wang S., Lou X., Xia F., Zhao Z., Tang B.Z. (2021). 9,10-Phenanthrenequinone: A Promising Kernel to Develop Multifunctional Antitumor Systems for Efficient Type I Photodynamic and Photothermal Synergistic Therapy. ACS Nano.

[29] Zhang P., Kuang H., Xu Y., Shi L., Cao W., Zhu K., Xu L., Ma J. (2020). Rational Design of a High-Performance Quinoxalinone-Based AIE Photosensitizer for Image-Guided Photodynamic Therapy. ACS Appl. Mater. Interfaces.

[30] Jadhao M., Ahirkar P., Joshi R., Kumar H., Ghosh S.K. (2016). Interaction of Aluminum Phthalocyanine with Aziridinyl Quinone in Biomimicking Micellar Microenvironment for the Application in Photodynamic Therapy: Effect of Micellar Hydration. J. Photochem. Photobiol. A Chem..

[31] Rodrigues B.M., Diniz C.C., da Rocha V.N., Köhler M.H., Brandão G.P., Machado L.A., da Silva Júnior E.N., Iglesias B.A. (2023). First Report of *Trans.*-A_2_B-Corrole Derived from a Lapachone Derivative: Photophysical, TD-DFT and Photobiological Assays. RSC Adv..

[32] da SS Campanholi K., Gerola A.P., Vilsinski B.H., de Oliveira É.L., de Morais F.A.P., Rabello B.R., Braga G., Calori I.R., Silva E.L., Hioka N. (2018). Development of Pluronic® Nanocarriers Comprising Pheophorbide, Zn-Pheophorbide, Lapachol and β-Lapachone Combined Drugs: Photophysical and Spectroscopic Studies. Dye. Pigment..

[33] Singh I., Ogata R.T., Moore R.E., Chang C.W.J., Scheuer P.J. (1968). Electronic Spectra of Substituted Naphthoquinones. Tetrahedron.

[34] Farfán R.A., Espíndola J.A., Gomez M.I., de Jiménez M.C.L., Martínez M.A., Piro O.E., Castellano E.E. (2012). Structural and Spectroscopic Properties of Two New Isostructural Complexes of Lapacholate with Cobalt and Copper. Int. J. Inorg. Chem..

[35] Segoloni E., Di Maria F. (2018). UV–VIS Spectral and GC–MS Characterization of Handroanthus Serratifolius (Vahl.) Grose (a.k.a. Tabebuia Serratifolia (Vahl.) Nichols/Lapacho) Heartwood Main Extractives: A Comparison of Protocols Aimed at a Practical Evaluation of Lapachol and Dehydro-α-Lapachone Content. Eur. J. Wood Wood Prod..

[36] Caires C.S.A., Silva C.M., Lima A.R., Alves L.M., Lima T.H.N., Rodrigues A.C.S., Chang M.R., Oliveira S.L., Whitby C., Nascimento V.A. (2020). Photodynamic Inactivation of Methicillin-Resistant Staphylococcus Aureus by a Natural Food Colorant (e-141II). Molecules.

[37] Nitzan Y., Gutterman M., Malik Z., Ehrenberg B. (1992). Inactivation of Gram-Negative Bacteria by Photosensitized Porphyrins. Photochem. Photobiol..

[38] Durantini E. (2006). Photodynamic Inactivation of Bacteria. Curr. Bioact. Compd..

[39] Caires C.S.A., Lima A.R., Lima T.H.N., Silva C.M., Araujo L.O., Aguilera L.F., Nascimento V.A., Caires A.R.L., Oliveira S.L. (2024). Photodynamic Inactivation of Methicillin-Resistant Staphylococcus Aureus by Using Giemsa Dye as a Photosensitizer. Photodiagnosis Photodyn. Ther..

